# Virotherapy: cancer gene therapy at last?

**DOI:** 10.12688/f1000research.8211.1

**Published:** 2016-08-30

**Authors:** Alan E. Bilsland, Pavlina Spiliopoulou, T. R. Jeffry Evans

**Affiliations:** 1 Institute of Cancer Sciences, University of Glasgow, Wolfson Wohl Cancer Research Centre, Glasgow, G61 1QH, UK; 2 Beatson West of Scotland Cancer Centre, Glasgow, G12 OYN, UK

**Keywords:** Cancer gene therapy, oncolytic virus, adenovirus, herpesvirus, cancer immunotherapy, Imlygic

## Abstract

For decades, effective cancer gene therapy has been a tantalising prospect; for a therapeutic modality potentially able to elicit highly effective and selective responses, definitive efficacy outcomes have often seemed out of reach. However, steady progress in vector development and accumulated experience from previous clinical studies has finally led the field to its first licensed therapy. Following a pivotal phase III trial, Imlygic (talimogene laherparepvec/T-Vec) received US approval as a treatment for cutaneous and subcutaneous melanoma in October 2015, followed several weeks later by its European authorisation. These represent the first approvals for an oncolytic virotherapy. Imlygic is an advanced-generation herpesvirus-based vector optimised for oncolytic and immunomodulatory activities. Many other oncolytic agents currently remain in development, providing hope that current success will be followed by other diverse vectors that may ultimately come to constitute a new class of clinical anti-cancer agents. In this review, we discuss some of the key oncolytic viral agents developed in the adenovirus and herpesvirus classes, and the prospects for further enhancing their efficacy by combining them with novel immunotherapeutic approaches.

## Introduction

In its broadest sense, the fundamental objective of cancer gene therapy is to transfer therapeutic transgenes specifically to cancer cells while leaving normal cells unharmed. In this paradigm, selectivity can be achieved at any or all of the levels of uptake, transgene expression, or intrinsic tumour sensitivities, and an enormous variety of constructs—coupled with diverse delivery approaches, including viral, bacterial, and chemical vectors—have now been investigated ^1^. The earliest approaches include gene replacement strategies involving the delivery of a wild-type tumour suppressor gene, which is either lost or deregulated in the target cell. An exemplar of this strategy is restoration of the p53 tumour suppressor that has been widely examined in a variety of preclinical models and clinical studies ^2– 6^.

Blocking the expression of activated oncogenes via antisense approaches has also been seen as attractive ^7^. However, the absence of positive results in early phase clinical trials has hindered further development of antisense nucleotides, and considerable interest is now focused on optimising oligonucleotide carrier formulations before embarking on further clinical studies ^8^. Other strategies for “suicide” or cytotoxic gene therapy include cell lysis to enhance tumour immunogenicity or the introduction of genes that amplify tumour sensitivity to drug or radiation therapies ^9^. These areas of gene therapy have been extensively discussed ^10^. Although measurable clinical activity has been found in a few “first-generation” approaches, such as adenoviral p53 replacement ^11^, highly promising results have recently been obtained utilising replicating viral vectors (oncolytic virotherapy) that help maximise gene transduction in tumour cells. In view of recent progress, we focus on this particular field of gene therapy.

The door for gene therapy in medicine was perhaps opened in November 2012, when the first human gene treatment, alipogene tiparvovec, was granted approval for patients suffering from familial lipoprotein lipase deficiency, a rare autosomal recessive disorder that leads to recurrent pancreatitis ^12^. Three years later, the approval of a virotherapy for cancer treatment marked a historic moment for cancer gene therapy in the Western world. The precedent has potentially profound importance for cancer therapeutics, since this approval is likely to represent only the first of a new and diverse class of agents. The concept of treating cancer with pathogenic organisms is now over a century old ^13^. However, the history of cancer virotherapy has followed cycles of interest and disappointment ^14^. In the years after the dawn of chemotherapy, as the first anti-cancer agents became available, numerous “oncolytic” viruses were tested for activity ^15^. Although interest waned after limited clinical successes were achieved, then-recent elucidation of the structure of DNA led even these early investigators to propose modifying viral genomes to rebalance oncolytic versus pathogenic properties ^15^.

Yet, since the first trial of viral-mediated gene transfer into patient tumours ^16^, realising the promise of gene therapy to provide an arsenal of exquisitely selective and potent anti-cancer agents has remained elusive until now. On 27 October 2015, Imlygic (talimogene laherparepvec/T-Vec) received full US Food and Drug Administration (FDA) approval for the treatment of melanoma, closely followed by European authorisation on 17 December. These approvals were a landmark moment, as the first anti-cancer gene therapy agent approved in the West entered into clinical practice.

The remarkable momentum of gene therapy in recent years has been extensively reviewed ^17– 20^. However, the most significant clinical success stories until now have remained in fields other than cancer. Many approaches suffer from limited vector penetration in tumours, which is often insufficient to produce substantial efficacy ^21^. Thus, conditionally replicating (oncolytic) vectors have been considered by many to be the best candidates for clinical success. Imlygic is an advanced-generation herpes simplex virus type 1 (HSV1) vector optimised for oncolytic activity and armed with the immunostimulatory granulocyte-macrophage colony-stimulating factor (GM-CSF) gene ^22^.

The immunomodulatory aspect of Imlygic activity may point the way to future successes; recently, growing awareness that anti-tumour immune responses mediate the efficacy of oncolytic agents has drawn cancer gene therapies and immunotherapies closer ^23– 26^. Indeed, several other oncolytic vectors armed with transgenes to stimulate anti-tumour T-cell responses are currently in development. In this context, another promising approach that has recently demonstrated remarkable clinical activity uses T-lymphocytes engineered with artificial chimeric antigen receptors (T-CARs). These may ultimately prove highly complementary to oncolytic agents.

## Oncolytic virotherapy and immunomodulation

Oncolytic virotherapy relies on selective replication of a virus specifically within cancer cells, triggering tumour cell death and vector spread into new cells. A wide range of vector backbones have been investigated, including “naturally oncolytic” organisms such as reovirus ^27– 29^. However, most agents comprise attenuated variants of well-characterised viruses. Here we focus on adenovirus and herpesvirus, since these backgrounds have undergone the most extensive vector engineering.

In these, selective replication is commonly achieved by the deletion of viral genes whose products ordinarily suppress cellular sentinels of the cell cycle, or of anti-viral responses. Replication is then facilitated in tumour cells with inactivation of these pathways; if such checkpoints are inactive, the requirement for a viral suppressor is removed. Alternatively, directing tumour-specific expression of the same viral genes using cancer-specific gene promoter elements achieves similarly restricted replication profiles via silencing viral protein expression in normal cells ^9, 30^.

Adenovirus replication depends initially on the expression of differentially spliced products of the early phase genes adenovirus early region 1A (E1A) and E1B, which together promote S-phase entry, setting the stage for the viral gene expression programme through multiple interactions with cellular transcription machinery. These include induction of transcription factor E2F by negative regulation of the retinoblastoma protein pRb by E1A and inhibition of checkpoint and apoptosis pathways by E1B ^31, 32^.

The earliest notable oncolytic adenovirus is dl1520/Onyx-015 ^33^. An Ad2/Ad5-hybrid lacking the E1B-55K gene, which acts in part through binding and inactivating p53, dl1520 was originally proposed to replicate selectively in tumour cells lacking p53 function and was originally developed by Onyx Pharmaceuticals (USA) ^34^. However, this mechanism was widely questioned, and subsequent investigations indicated that late functions of E1B-55K, involving the regulation of translation, are rate limiting for dl1520 replication ^35^.

The agent was safely delivered via both intratumoural ^36– 40^ and intravascular (mainly hepatic arterial) or intraperitoneal routes ^38, 41, 42^, targeting either primary or secondary malignant hepatic disease. As monotherapy, dl1520 showed modest clinical outcomes, mainly in the form of disease stabilisation; however, when combined with cytotoxics in head and neck tumours and colorectal liver metastases, it conferred re-sensitisation to chemotherapy against which these tumours had previously shown resistance ^39, 41, 42^. Correlation between p53 mutation status and response to treatment was not shown, however, amplifying uncertainties regarding the virus’ mechanism of action. The company halted clinical development in 2003; however, the highly related vector H101 was licensed to Sunway Biotech (China) and was approved for use there in 2005.

Improvements to replicating adenovirus design have since accumulated, including enhanced strategies for restricting replication and modifications to vector tropism. DNX-2401 (DNAtrix Therapeutics [USA]), formerly known as Ad∆24-RGD, is a second-generation Ad5 vector based on an alternative replication-targeting approach. In the original vector, Ad∆24, the viral E1A gene, reintroduced into a first-generation (E1/E3 deleted) backbone, harbours a 24 bp deletion in the pRb binding site in order to restrict efficient replication to cells with a defective pRb\p16\E2F pathway ^43^. Subsequently, tropism of the vector was expanded in Ad∆24-RGD, which contains a short peptide harbouring an integrin-binding RGD motif in the viral receptor-binding protein, known as fibre ^44^.

Ad5/Ad2 internalisation involves high-affinity interaction between the terminal “knob” domain of the trimeric fibre and the primary cellular receptor, human coxsackie and adenovirus receptor (hCAR) ^45– 47^. Subsequently, interactions between cellular αvβ3/αvβ5 integrins and RGD sequence motifs in the capsid protein penton mediate endocytosis ^48, 49^. Hence, modifications to fibre can target Ad2/Ad5 to alternative receptors. hCAR expression is low in some cancer cells and it can also be sequestered in tight junctions between epithelial cells ^50– 52^. RGD modification provides a tropism extension by re-directing high-affinity binding to integrins.

An alternative approach to tropism modification is “pseudotyping”; here, the vector knob domain is wholly replaced by that from a different adenovirus serotype, exhibiting different tropism. For example, Ad3 utilises an alternative receptor to Ad2/Ad5 ^53^. Ad5∆24 vectors expressing the Ad3 knob efficiently infected and replicated in ovarian cancer cells that were resistant to vectors expressing wild-type Ad5 knob ^54^. Oncos-102/CGTG-102 (Oncos Therapeutics [Finland]) contains the E1A-∆24 mutation, is pseudotyped with Ad3 knob, and is also armed by the addition of the GM-CSF gene in the deleted E3 region in order to promote CD8+ T-cell responses against infected cells.

An early trial of CGTG-102 showed that intratumoural or intracavitary delivery in heavily pre-treated patients induced disease responses even when given as a single dose. Furthermore, the vector induced distant anti-tumour immunity ^55^. After demonstrating 63% disease stabilization in 16 patients, treatment was expanded to 115 trial patients. Serial treatment with CGTG-102 resulted in significantly improved survival (p<0.0001) when compared to single dosing and confirmed the safety of repeat dosing ^24^. Efficacy results were assessed radiologically, and the patients deriving most benefit were those with soft tissue sarcoma, ovarian cancer, melanoma, mesothelioma, and breast cancer ^56^. Further improvements to the replication targeting of Ad5∆24 vectors have been made via the third-generation ICOVIR vectors, which combine E1A mutation with tight transcriptional control and translational optimisation ^57^.

Most adenoviral vectors are based on well-characterised laboratory strains representing a restricted range of serotypes. ColoAd1 is a chimeric virus, generated through selection by “directed evolution”, whereby a pool of Ad serotypes from groups B-F are passaged through cell lines of breast, colon, prostate, and pancreatic cancer to allow recombination of potent viral serotypes. ColoAd1 “emerged” through a colon cell line (HT-29)-passaging pool, and is a chimera of Ad11 and Ad3 serotypes belonging to adenovirus Group B ^58^. When tested on a colon cancer liver-seeding model, it demonstrated increased anti-tumour potency *in vivo* compared to both Ad5 and dl1520.

The group B origin of Colo-Ad1 gives the distinct advantage of an hCAR-independent attachment to cells via principal binding to CD-46 receptor, which is expressed by a variety of tumours such as thyroid, breast, ovarian, endometrial, lung, colorectal, pancreatic, and gastric, and is amplified in higher grade tumours ^59, 60^. Proof-of-concept studies are ongoing in bladder/colorectal (NCT02028442) and ovarian (NCT02028117) cancer patients via both systemic and intraperitoneal routes, whilst the combination of ColoAd1 with inhibition of the PD1/PDL1 axis is also planned (NCT02636036).

The herpesviruses present a second major vector background that has undergone significant development. HSV1716 (SEPREHVIR, Virttu Biologics [UK]) is an ICP34.5-deleted first-generation oncolytic HSV1 vector. A 759 bp deletion, which conferred avirulence on intracerebral inoculation, was originally identified in a variant of the HSV1 17+ strain. HSV1716 was developed by re-introduction of this spontaneously arising deletion into the wild-type 17+ background ^61^. The vector was found to replicate selectively in dividing cells, causing cytotoxicity to tumour cells and regression in xenograft models ^62, 63^. Safety was shown following direct intratumoural injection in glioma patients ^64, 65^ and in the intraoperative setting of injection to tumour-adjacent brain tissue after debulking ^66^.

G207 is a second-generation HSV1 vector based on strain F, containing deletions in both loci of ICP34.5 in addition to inactivation of ICP6, thereby disabling the viral ribonucleotide reductase ^67^. The vector also showed safety in phase I trials in glioblastoma: initially, a single inoculation dose escalation study was performed ^68^. Subsequently, the vector was tested in a intraoperative setting (pre- and post-resection) and in combination with radiotherapy ^69, 70^. A further trial (NCT02457845) is planned to assess the safety of G207 alone or with radiation in paediatric patients, but it is not yet recruiting. NV1020 (R7020), another multiply deleted vector based on strain F, contains a 15 kb deletion of the “joint region”, which includes a single copy of ICP34.5, in addition to the U _L_56 gene. This vector is less attenuated than G207.

As noted above, Imlygic is the first oncolytic vector to receive approval in the US and Europe. It is an HSV1 vector optimised in several ways ^22^. The parental strain JS1 was obtained from a new clinical isolate rather than the serially passaged laboratory strains previously utilised in HSV1716 and G207. Unattenuated JS1 demonstrated significantly higher cytotoxic activity than the wild-type 17+ strain in several tumour cell lines. The oncolytic vector was generated by deletion of the ICP34.5 gene in addition to ICP47, which is involved in suppressing antigen presentation ^71^. Loss of ICP47 therefore promotes an immune response against infected tumour cells, and this aspect is further enhanced through arming with the GM-CSF gene.

The OPTiM trial was the first randomised controlled phase III study of an oncolytic agent to have met its efficacy end-point, and thus enough supporting evidence was provided for its recent approval ^72^. Patients with unresected stage III or IV melanoma and with variable lines of previous treatments were randomised to either intralesional treatment of Imlygic, or systemic treatment with GM-CSF. Clinical efficacy was confirmed with 26.4% of patients experiencing an overall response (complete response [CR] or partial response), and in 16.3% of patients this response lasted for more than 6 months.

With a much more favourable toxicity profile than that observed using current immune checkpoint inhibitors, Imlygic also achieved a higher rate of CRs (10% CR rate versus the historic 1–6% rate observed with ipilimumab and pembrolizumab) ^73, 74^. Both the treatment responses and the survival advantage achieved with intralesional Imlygic were statistically significant in patients with earlier stage than M1b/M1c (without visceral metastases or raised lactate dehydrogenase) with a hazard ratio (HR) for overall survival of 0.57 (p<0.001) when compared to treatment with GM-CSF. This effect should not be attributed only to intralesional oncolysis, as 15% of uninjected visceral lesions reduced their size by ≥50%. Greater benefit, in terms of both response and survival, was also observed in treatment-naïve patients (HR 0.50, p<0.001). The study design minimised treatment discontinuations due to misperceived “pseudo-progressions” allowing patients to be treated for a minimum of 24 weeks if clinically appropriate. Overall, the results confirm the activity of Imlygic in a subset of patients with low-volume injectable melanoma not subjected to multiple lines of treatment. However, it should be noted that systemic GM-CSF is a comparator that lacks a statistically confirmed impact on overall survival ^75^. Therefore, further studies are needed to validate the role of Imlygic in the melanoma therapeutic algorithm.

How can the cancer gene therapy field now build on this success? The development of several agents has previously been incentivised via orphan drug designations ( Table 1). Most have not yet progressed to pivotal phase III trials, although DNX-2401 may do so in 2016 ( http://www.dnatrix.com/pipeline/). The approval of Imlygic increases confidence that more approvals could be achieved for vectors that “stay the course”. The wide variety of vector backgrounds being tested is also cause for optimism; this medley of tropisms, lytic cycles, and immunological effects ensures that the agent class is highly diverse.

**Table 1.** Oncolytic vectors granted US Food and Drug Administration (FDA) orphan drug designation.

Agent	Vector	Company	Disease	Designation year
G207	Herpes simplex virus	Aettis, Inc.	Glioma	2002
NTX-010	Seneca Valley Virus	Neotropix	Neuroendocrine tumours	2008
ONCOS-102	Adenovirus	Oncos Therapeutics	Malignant mesothelioma, ovarian cancer, and glioma	2013–14
DNX-2401	Adenovirus	DNAtrix	Glioma	2014
Reolysin	Reovirus	Oncolytics Biotech Inc.	Glioma, gastric cancer, primary peritoneal cancer, fallopian tube cancer, ovarian cancer, and pancreatic cancer	2015

Imlygic continues development in a number of early phase trials for other solid tumours including pancreatic adenocarcinoma, soft tissue sarcoma, and head and neck squamous cell carcinoma ^76^. Furthermore, there may be considerable advantage in combining oncolytic agents with other immunomodulatory strategies. ** Combination approaches utilising immune checkpoint inhibitors ipilimumab and pembrolizumab are currently being tested, reflecting a view that efficacy gains could be made through further stimulation of anti-tumour immunity beyond those mechanisms inbuilt in the vector. Preliminary results support a possible synergistic effect in treatment-naïve melanoma patients using Imlygic as a priming agent before immune-induction with ipilimumab ^77^.

Many mechanisms allow tumours to evade natural immunity that would otherwise recognise and eliminate cancer cells ^78^. Cancer immunotherapy uses various approaches to overcome immune tolerance. In particular, the use of T-cells specifically targeted to tumours has shown considerable clinical promise in recent years. Cytotoxic CD8+ T-cells isolated from cancer patients can recognise tumour-associated antigens via the major histocompatibility complex class I antigen presentation pathway ^79^. However, *in vivo*, their anti-tumour activities are blunted. Clinical approaches to enhance T-cell responses have included *ex vivo* stimulation of antigen-presenting cells with tumour-derived antigens or mRNA ^80^, systemic administration of synthetic peptides capable of binding class I molecules ^81^, and pharmacological immune-checkpoint inhibitors ^82^.

An alternative approach circumventing the requirement for antigen processing and presentation involves T-cells transduced *ex-vivo* with chimeric antigen receptors (T-CARs). The synthetic receptors commonly comprise single-chain antibody fragments serving as the extracellular antigen-recognition domain fused to the CD3ζ transmembrane adaptor signalling domain, with or without additional co-stimulatory domains ^83^. Together, these serve to signal T-cell activation on binding to cell-surface antigens. T-CARs targeted to CD19 have recently demonstrated significant clinical response rates in patients with haematological malignancies, particularly chronic lymphocytic leukaemia ^84– 87^. Indeed, sustained responses of several-year duration have been reported in some patients ^88^. T-CARs are also being developed against a wide range of solid-tumour antigens ^89^.

A particularly interesting avenue for future trials may therefore lie in combining armed immunomodulatory oncolytic agents with T-cell targeting, potentially also alongside immune-checkpoint inhibition. It was recently demonstrated that T-CARs targeted to human epidermal growth factor receptor 2 could act as effective carriers of oncolytic vaccinia or vesicular stomatitis viruses ^90^. Neither the oncolytic passengers nor the T-cell vehicles appeared to significantly interfere with each other’s activities. However, T-cells are non-permissive for infection by certain vectors, including commonly used adenovirus serotypes, because of low viral receptor expression ^91^. Nevertheless, alternative approaches could still allow these modalities to be combined: in another study, local delivery to neuroblastoma xenografts of Ad5Δ24 armed with cytokines RANTES and IL15 enhanced infiltration and persistence within the tumour of subsequently delivered T-CARs targeted to the GD2 antigen ^92^.

## Outlook

There is considerable scope for multi-modal immunogenetic therapies to improve further on emerging successes. However, the approval path for advanced biotherapies is not necessarily straightforward ^93– 96^. Various guidelines have been developed to clarify requirements for viral vector programmes ^97, 98^. However, to streamline the translation of potentially promising new agents, it is important that researchers adopt a future-focused approach. A recent manifesto calls for a range of practical measures to be embedded in gene therapy programmes. These include using well-defined target-product profiles (as in Pharma), establishing ambitious pre-clinical efficacy cut-offs, planning early for phase I-III clinical studies, and, critically, planning for manufacture and scale-up ^99^.

These general principles should be embedded in programmes and may ease clinical development, but we believe that effective trial design is the core of the issue; fundamentally, late-phase trials must achieve their efficacy endpoints. It remains to be seen if oncolytic vectors can be developed that will be effective when administered systemically. Therefore, delivery routes may continue to dictate those tumours that are tractable. Imlygic is delivered by direct intratumoural injection to cutaneous lesions; intraperitoneal delivery is also an attractive localised route in the setting of ovarian cancer ^100^.

Another key aspect of trial design for gene therapy relates to stratification and pharmacodynamic biomarkers, which will likely prove increasingly critical to the development of gene therapy agents, as for other cancer therapeutics. The absence of suitable stratification markers necessarily leads to a requirement for larger patient groups to identify robust responders. This is clearly undesirable, given that viral vector manufacture and scale-up involve so many variables ^101^. Indeed, infectivity and growth characteristics of oncolytic agents are individually tailored such that process standardisation for any “vector class” is likely to be problematic. These aspects raise difficulties in predicting production requirements for viral gene therapy regimens, issues that will be compounded in moving to larger efficacy trials. For oncolytics, increasing evidence for the role of anti-tumour immunity in mediating efficacy is leading to the investigation of a range of candidate predictive immunological markers ^23, 24, 26^. On the other hand, given the still-partial mechanistic understanding of some agents, the optimal choice of biomarkers may emerge only during development.

Yet, though the hurdles may be higher, the road to effective gene therapies should be seen in the context of the success rate throughout cancer drug development, even for small molecules ^102^. Taking an optimistic outlook, if the long process of vector development that led us to this point is viewed analogously to lead optimisation in traditional drug development, then gene therapists do not, at least, need to begin with a new scaffold for each target, unlike medicinal chemists. It is fair to suggest that the wilderness years of gene therapy are in the past and that once the optimal vector configurations for cancer applications are understood they will be “selectively replicated”. By combining these with innovative new arming approaches and other modalities, the legacy of Dock ^1^ and others may finally be realised.

## Abbreviations

CR, complete response; E1, adenovirus early region 1; GM-CSF, granulocyte-macrophage colony-stimulating factor; hCAR, human coxsackie and adenovirus receptor; HR, hazard ratio; HSV1, herpes simplex virus type 1; T-CARs, chimeric antigen receptor-engineered T-cells.
